# Single-institution Retrospective Analysis of Prognostic Factors Influencing Very Late-onset Post-transplant Lymphoproliferative Disorder

**DOI:** 10.7759/cureus.6912

**Published:** 2020-02-07

**Authors:** Rohit Bishnoi, Jordan Minish, Aaron J Franke, William P Skelton, Chintan P Shah, Yu Wang, Nam H Dang

**Affiliations:** 1 Hematology and Oncology, University of Florida, Gainesville, USA; 2 Internal Medicine, University of Florida, Gainesville, USA; 3 Oncology, H. Lee Moffitt Cancer Center and Research Institute, Tampa, USA; 4 Health Cancer Center, University of Florida, Gainesville, USA; 5 Oncology, University of Florida, Gainesville, USA

**Keywords:** post transplant lymphoproliferative disorder, late-onset ptld, very late-onset ptld, prognostic factors for ptld, ebv ptld

## Abstract

Background

Post-transplant lymphoproliferative disorder (PTLD) is a rare complication following transplant (solid organ or allogeneic) due to the proliferation of lymphoid cells in the immunosuppressed state. The incidence of PTLD follows a bimodal distribution, with high incidence immediately after transplant (early-onset PTLD), followed by a decline and then a high-incidence again five years after transplantation (late-onset PTLD). This study exclusively aims to identify prognostic factors for the subgroup of PTLD, described as very late-onset PTLD, occurring after 10 years of transplant.

Methods

This study was conducted at the University of Florida, with the requisite study population identified through the cancer registry. Data were collected by individual chart review and analyzed. Survival estimates and univariate and multivariate analyses were performed to measure the effects of each variable on overall survival.

Results

A total of 33 patients were identified, with a median age at transplant of 42.3 years, while the median age at PTLD diagnosis was 54.7 years. Median time from transplant to PTLD diagnosis was 13.3 years. Kidney (30.3%), liver (27.3%), and heart (24.2%) transplants were the most common allografts associated with very late PTLD development. The most common pathology was diffuse large B-cell lymphoma (DLBCL) in 45.5% of patients. CHOP+/-R (cyclophosphamide, doxorubicin hydrochloride (hydroxydaunorubicin), vincristine sulfate (Oncovin), prednisone, rituximab) was the most common chemo regimen used as the initial choice in 36.4% of patients.

Median survival was 5.4 years. Univariate analysis showed that age at diagnosis over 65, male gender, bone marrow involvement, past medical history (PMH) of malignancy, immunosuppression regimen at PTLD diagnosis, and initial and final best response to treatment were statistically significant (p <0.05) factors associated with survival. On multivariate analysis, bone marrow involvement was significantly associated with poor survival (p=0.008). Surprisingly, performance status, Epstein-Barr virus (EBV) status, pathology type, Ann-Arbor stage, and chemotherapy regimen were not significantly associated with survival. At the end of the study, 48.5% of patients achieved complete remission and the allograft survived in 84.8%.

Conclusions

In this retrospective study of very-late onset PTLD, we identified factors associated with survival different from early and late PTLD. These factors should be considered during the treatment of this subgroup of PTLD patients.

## Introduction

Post-transplant lymphoproliferative disorder (PTLD) describes the process of the proliferation of lymphoid cells in the immunocompromised state caused by immunosuppressive agents used after the transplant of solid organ or allogeneic stem cells. It was first described in solid organ transplant patients by Doak et al. in 1968 and is a well-documented, albeit rare, complication. It is the leading cause of cancer-related mortality following transplant [1]. The incidence of PTLD is higher after intestinal or multiorgan transplants and the lowest after liver or hematopoietic stem cell transplants [2-3]. PTLD incidence has been on the decline over the last few decades, with some studies reporting a decline in incidence from more than 20% to less than 3% in a period ranging from 1990 to 2011 [4]. Numerous risk factors place a patient at a higher likelihood of developing PTLD, most importantly, the degree of immunosuppression of T cells and recipient Epstein-Barr virus (EBV) status [5]. Older age, white race, and time elapsed after transplant have also been described as risk factors [4]. Various single-center institutional studies have analyzed and reported outcomes for PTLD, which remains the main source of clinical information on this rare entity [6-9]. Being one of the largest transplant centers in the southeast United States, we analyzed the outcomes of PTLD at our institution [10-12].

In the 2008 World Health Organization (WHO) classification of lymphoma, PTLD was recognized as a separate group of lymphoid malignancies, where PTLD was classified into early-lesion PTLD, polymorphic PTLD, monomorphic PTLD, and classical Hodgkin lymphoma PTLD. The incidence of PTLD follows a bimodal distribution, with a higher incidence immediately after transplant, followed by a decline, and then a high-incidence again five years after transplantation and again extending to more than 10 years after transplant [4,13-15]. This incidence pattern, depending on the time interval between transplant and PTLD diagnosis, has led to another classification of PTLD in the medical literature, early-onset and late-onset PTLD. PTLD occurring within 12 months of transplant is defined as early-onset while PTLD developing after 12 months is described as late-onset PTLD [4,8,16-17]. These two entities have been described to have different risk factors and clinicopathologic features.

Early-onset PTLD is more frequently associated with EBV viremia, CD20 positivity, young age, and allograft involvement while late-onset PTLD is associated more with advanced age, EBV seronegativity, induction therapy with polyclonal antibodies or OKT3, and azathioprine therapy, and has more frequent extra-nodal disease [4,8,17-20]. Recipient EBV seronegativity does increase the risk of early and EBV positive PTLD [4,20]. Also, late-onset PTLD tends to be more B-cell in origin and monomorphic pathology, less likely to involve allograft and tends to resemble more with lymphoma in the general population [18,21]. Due to the role of EBV in the pathogenesis of PTLD, especially for early-onset PTLD, EBV viral load monitoring has been utilized by certain institutions as a strategy to decrease PTLD risk, but this has not shown a significant impact on PTLD risk reduction [20]. Viral load monitoring and close follow-up after transplant do help in identifying early PTLD.

The incidence of PTLD has been on the decline but due to the improved survival of grafts with longer immunosuppression, we continue to see new cases of PTLD even a long time after organ transplantation [22-23]. It is important to gather more scientific evidence regarding prognostic factors for late-onset PTLD, as previous studies have shown that these patients tend to have poor survival [4,24-25]. There is another subset of PTLD occurring after a decade of transplant, which has been described in medical literature as ‘very-late’ onset PTLD [21-22]. Very little is known about factors affecting survival in this subgroup of PTLD, but it has been reported to mainly affect older patients [21,26]. Over the last many years, multiple studies have focused on early versus late-onset PTLD, with limited data about PTLD occurring after a decade of transplant. Our literature search showed that apart from a few isolated case reports, only one retrospective study focused on this subgroup [21]. With this study from a large single institution, we aimed to examine prognostic factors influencing the outcomes of ‘very-late onset PTLD.’ To our knowledge, this is the first-ever retrospective single institutional study reported from the United States specifically targeting this subgroup of very late-onset PTLD.

## Materials and methods

This study was conducted at the University of Florida after institutional review board (IRB) approval and all ethical guidelines were followed.

The study population was identified through the cancer registry at the University of Florida (UF) Health Cancer Center. These patients were diagnosed with PTLD from April 2003 to December 2016 over a span of 14 years. Data were collected by individual chart review. Data collected included patient demographics, transplant, PTLD details, treatments, and outcomes. Data were analyzed as percentages for each variable and survival estimates; univariate and multivariate analyses were performed to measure the effects of each variable on overall survival.

Treatment response was defined as complete response (CR), partial response (PR), stable disease (SD), and progressive disease (PD). The overall response rate (ORR) was defined as CR+PR while the disease control rate (DCR) was defined as CR+PR+SD.

Statistics method

Descriptive statistics were calculated for all variables, including the frequency of nominal variables and mean, median, standard deviance of numeric variables. All hazard ratios (HRs) and their 95% confidence interval were estimated from Cox proportional hazard (CoxPH) models. The Kaplan-Meier method was applied to fit the overall survival functions. Then, the Log-rank test was used to compare the survival curves between subgroups.

In order to identify the risk factors that were statistically associated with overall survival, univariate and multivariate CoxPH models were performed sequentially. Univariate analysis was performed using variables, including patient characteristics, transplant details, time from transplant to PTLD, immunosuppression details, PTLD characteristics, and treatment details among others. All variables with a p-value of HR less than 0.2 in univariate analysis were selected to enter the multivariate CoxPH model as covariates. Then, a stepwise selection method with an entering p-value of 0.2 and a staying p-value of 0.05 was applied to do the model section.

Without a special remark, all statistically significant levels were defined as p < 0.05. Data analysis was conducted with SAS 9.4 (SAS Institute, Cary, North Carolina).

## Results

A total of 33 patients were identified who developed PTLD after 10 years of transplant. The median age at transplant was 42.3 years (range: 2 months - 64.6 years) while the median age at PTLD diagnosis was 54.7 years (range: 11.2 - 79.4 years). Median time from transplant to PTLD diagnosis was 13.3 years (range: 10 - 18.4 years). Other demographic details are listed in Table 1.

**Table 1:** Patient demographics and PTLD details with HR for overall survival EBER: Epstein-Barr encoding region, NSS: not statistically significant, IPI: International Prognostic Index, ECOG: Eastern Cooperative Oncology Group, PMH: past medical history; PTLD: Post-transplant lymphoproliferative disorder; HR: hazard ratio

Variables		n	%	Hazard Ratio	p-value
Sex					
	Male	19	57.6	6.1	0.0189
	Female	14	42.4		
Race				NSS	
	White	23	69.7		
	Black or African American	4	12.1		
	Asian	3	9.1		
	Other	3	9.1		
Age group at diagnosis					
	Pediatrics	5	15.2		
	Adults	21	63.6		
	Elderly	7	21.2	3.9	0.0267
Induction immunosuppression				NSS	
	Yes	8	24.2		
	No	21	63.6		
	UK	4	12.1		
Allograft type				NSS	
	Kidney	10	30.3		
	Liver	9	27.3		
	Heart	8	24.2		
	Lung	4	12.1		
	Kidney and pancreas	2	6.1		
Acute allograft rejection before PTLD				NSS	
	Yes	16	48.5		
	No	16	48.5		
	Unknown	1	3.0		
Tumor EBER status				NSS	
	Positive	9	27.3		
	Negative	18	54.5		
	Unknown	6	18.2		
ECOG status at PTLD diagnosis				NSS	
	0-2	28	84.8		
	3-4	4	12.1		
	Unknown	1	3.0		
CD 20				NSS	
	Positive	21	63.6		
	Negative	8	24.2		
	Unknown	4	12.1		
Extra-nodal sites				NSS	
	Positive	19	57.6		
	Negative	11	33.3		
	Unknown	3	9.1		
Ann-Arbor Stage				NSS	
	I-II	16	48.5		
	III-IV	15	45.5		
	Unknown	2	6.1		
Albumin level					
	Normal	19	57.6	0.35	0.0760
	Low	12	36.4		
	Unknown	2	6.1		
IPI score				NSS	
	0-2	15	45.5		
	3-5	10	30.3		
	Unknown	8	24.2		
PMH of malignancy				4.8	0.0481
	Yes	2	6.1		
	No	31	93.9		
B-symptoms				NSS	
	Yes	13	39.4		
	No	17	51.5		
	Unknown	3	9.1		

Twenty-four point two percent (24.2%) (n=8) patients received induction immunotherapy and the most commonly used drug was OKT3. The most common initial immunosuppression regimen used after transplant was steroids, cyclosporine, and azathioprine in 51.5% (n=17) patients. At the time of PTLD diagnosis, tacrolimus alone or in combination with mycophenolate or azathioprine was the most common agent used for immunosuppression in 51.5% (n=17) patients.

Donor EBV serostatus data was mostly not available. Recipient EBV serostatus was positive in 45.5% (n=15) patients before transplant and was positive in 54.5% (n=18) after transplant. The tumor EBV-encoded RNA (EBER) was positive only in 27.3% (n=9) patients. Over the course of more than 10 years after transplant, 48.5% (n= 16) experienced acute rejections of allograft and 21.2% (n=7) experienced more than one episode of acute rejection. Chronic allograft rejection was noted in 21.2% (n=7) patients.

B symptoms were present in 48.5% (n=16) patients and weight loss was the most common symptom present in 30.3% (n= 10). Six point one percent (6.1%) (n=2) patients had a past medical history of other unrelated malignancies.

Monomorphic PTLD was noted in 72.8% (n=24) patients and diffuse large B-cell lymphoma (DLBCL) was the most common pathologic subtype noted in 45.5% (n=15). Detailed pathologic distribution is outlined in Table 2.

**Table 2:** PTLD pathologic distribution DLBCL: diffuse large B-cell lymphoma, HL: Hodgkin like; PTLD: post-transplant lymphoproliferative disorder

PTLD Pathology	Subtypes	B-Cell Subtype	n	%
Early lesion PTLD				
	Plasmacytic hyperplasia		1	3.0
Polymorphic PTLD			6	18.2
Monomorphic PTLD				
	B-Cell type			
		DLBCL	15	45.5
		Burkitt’s	1	3.0
		Plasma cell myeloma or plasmacytoma like lesion	3	9.1
		Unspecified B-cell	3	9.1
	T-cell type		2	6.1
Hodgkin or HL like PTLD			2	6.1

Table 3 shows the details of upfront therapy. As upfront therapy, 15.2% (n=5) patients were treated with reduced immunosuppression (RIS) alone and 3% (n=1) was treated with surgery alone. Eighty-one point eight percent (81.8%) (n=27) received upfront chemotherapy, either rituximab alone or in combination with other chemo drugs. Another 6.1% (n=2) patients received chemo after the failure of upfront therapy and thus a total of 87.9% (n=29) patients required chemotherapy for PTLD treatment.

**Table 3:** Treatment response after upfront therapy and at the end of the study CR: complete remission; PR: partial remission; SD: stable disease; PD: progressive disease; DCR: disease control rate; ORR: overall response rates

Response	Initial Response		Final Response	
	n	%	n	%
CR	17	51.5	16	48.5
PR	5	15.2	1	3.0
SD	2	6.1	4	12.1
PD	5	15.2	7	21.2
Unknown	4	12.1	5	15.2
DCR	24	72.7	21	63.6
ORR	22	66.7	17	51.5

Rituximab alone as upfront therapy was used in 18.2% patients (n=6) and of these six patients, three received further chemotherapy in combination with rituximab. CHOP+/-R was the most common chemo regimen used as an initial choice in 36.4% (n=12) patients. Another 9.1% (n=3) received CHOP+/-R as subsequent therapy after the failure of upfront therapy. During PTLD treatment, overall 45.5% (n=15) received CHOP+/-R, which was the most commonly used regimen. This was followed by R/C (rituximab, cyclophosphamide) and R-CVP (rituximab, cyclophosphamide, vincristine​, prednisone) in 9.1% (n=3) each.

At the end of the study, 48.5% (n=16) had a complete response and the overall response rate was 51.5% (n=17). Table 4 outlines the details of the response to upfront therapy and at the end of treatment.

**Table 4:** Treatment details of upfront therapy RIS: reduced immunosuppression

Upfront Therapy		n	%
RIS alone		5	15.2
Chemotherapy		27	81.8
	Rituximab	6	18.2
	R+Chemo	17	51.5
	Chemo	4	12.1
Surgery		1	3.0

At the end of the study, 54.5% (n=18) patients were alive and of these, 13 patients were alive with PTLD while 39.4 (n=13) patients had deceased. After PTLD diagnosis, the allograft survived in 84.8% (n=28) patients. Six point one percent (6.1%) (n=2) patients eventually lost their graft after the diagnosis of PTLD and one of those patients was re-transplanted later, without any issues. One patient experienced allograft rejection during PTLD treatment but was salvaged by the use of pulse steroids and increased immune-suppression. Table 5 gives details of the final outcomes of patients and allografts.

**Table 5:** Final outcomes PTLD: post-transplant lymphoproliferative disorder

Outcome	Variable		n	%
Patient				
	Alive		18	54.5
		Alive with PTLD	13	39.4
		Alive without PTLD	5	15.2
	Dead		13	39.4
		From PTLD	3	9.1
		Unrelated cause	3	9.1
		Unknown cause	7	21.2
	Unknown, if alive or dead		2	6.1
Graft	Graft survived		28	84.8
	Graft failed		2	6.1
	Unknown		3	9.1

Statistical analysis

**Figure 1:**
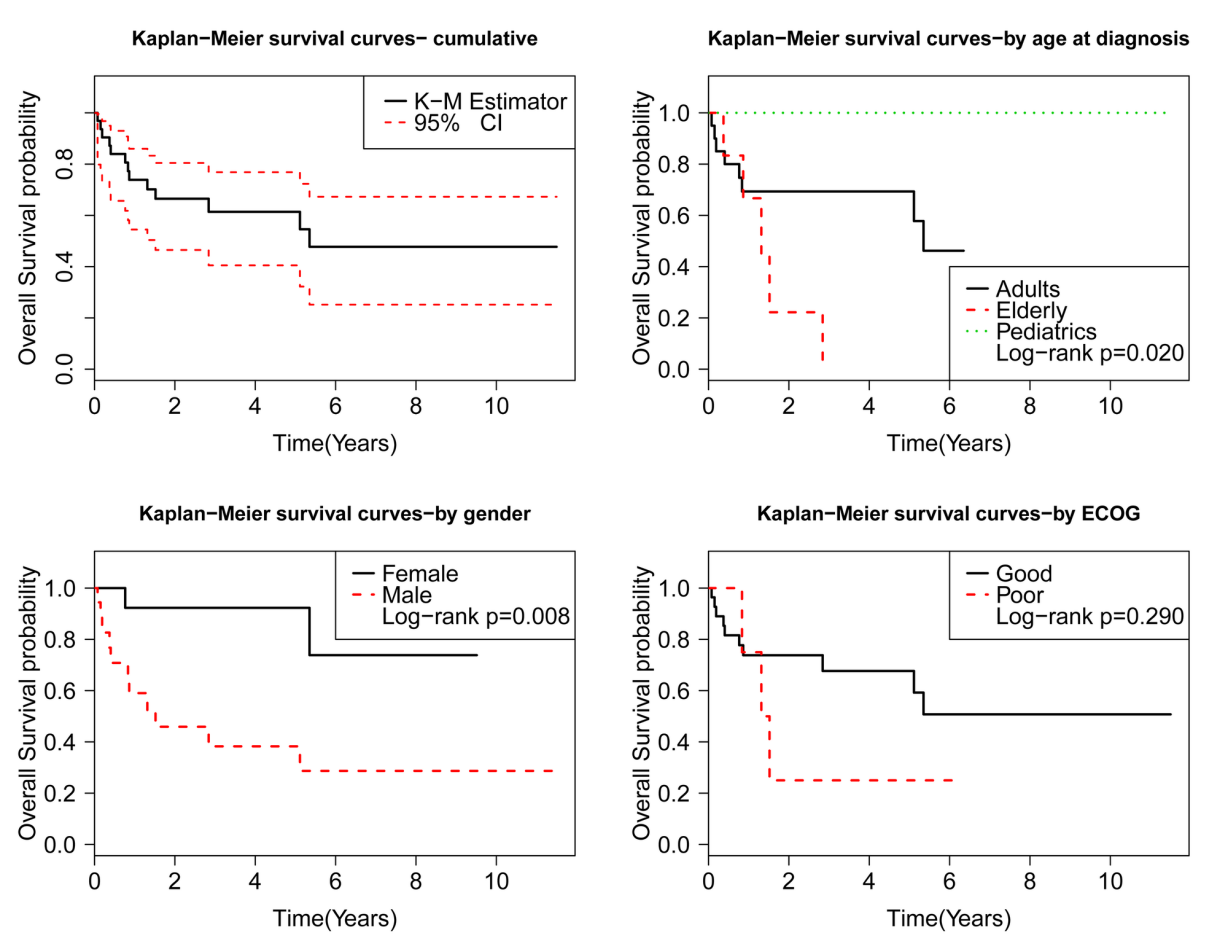
Kaplan-Meier survival analysis based on patient demographics

The Kaplan-Meier (KM) analysis showed a median overall survival time of 5.4 years. Univariate analysis was performed to identify factors associated with survival. The results showed the following patient characteristics to be statistically significant for survival: age at diagnosis of >65 years had HR of 3.9 (95% CI: 1.2-13.1, p=0.027), male gender had HR of 6.1 (95% CI: 1.3-27.6, p=0.019), and PMH of malignancy had HR of 4.8 (95% CI: 1.0-22.8, p=0.048). Recipient EBV and CMV status were found to be non-significant. The patient’s race, ECOG, International Prognostic Index (IPI) score, time from transplant to PTLD diagnosis, type, and previous rejection episodes were non-significant for overall survival. See Figure 1.

Analysis of immunosuppression drugs being used at the time of PTLD diagnosis showed that the combination of cyclosporine with azathioprine had HR 5.7 (95% CI: 1.8-18.4, p= 0.003). If tacrolimus was used in combination or by itself as an immunosuppressant, patients had HR of 0.13 (95%CI: 0.03-0.57, P= 0.007).

Analysis of the best treatment response showed that patients with an initial best response of PD had an HR of 6.3 (95%CI: 1.4-28.1, p=0.01); similarly, patients with the final response as PD had HR: 8.0 (95% CI: 1.9-34.1, p=0.005). Whereas patients who achieved CR as the final response had an HR of 0.2 (95% CI: 0.1-0.9, p=0.035). The use of rituximab or a different chemotherapeutic regimen was not statistically significant for survival.

**Figure 2:**
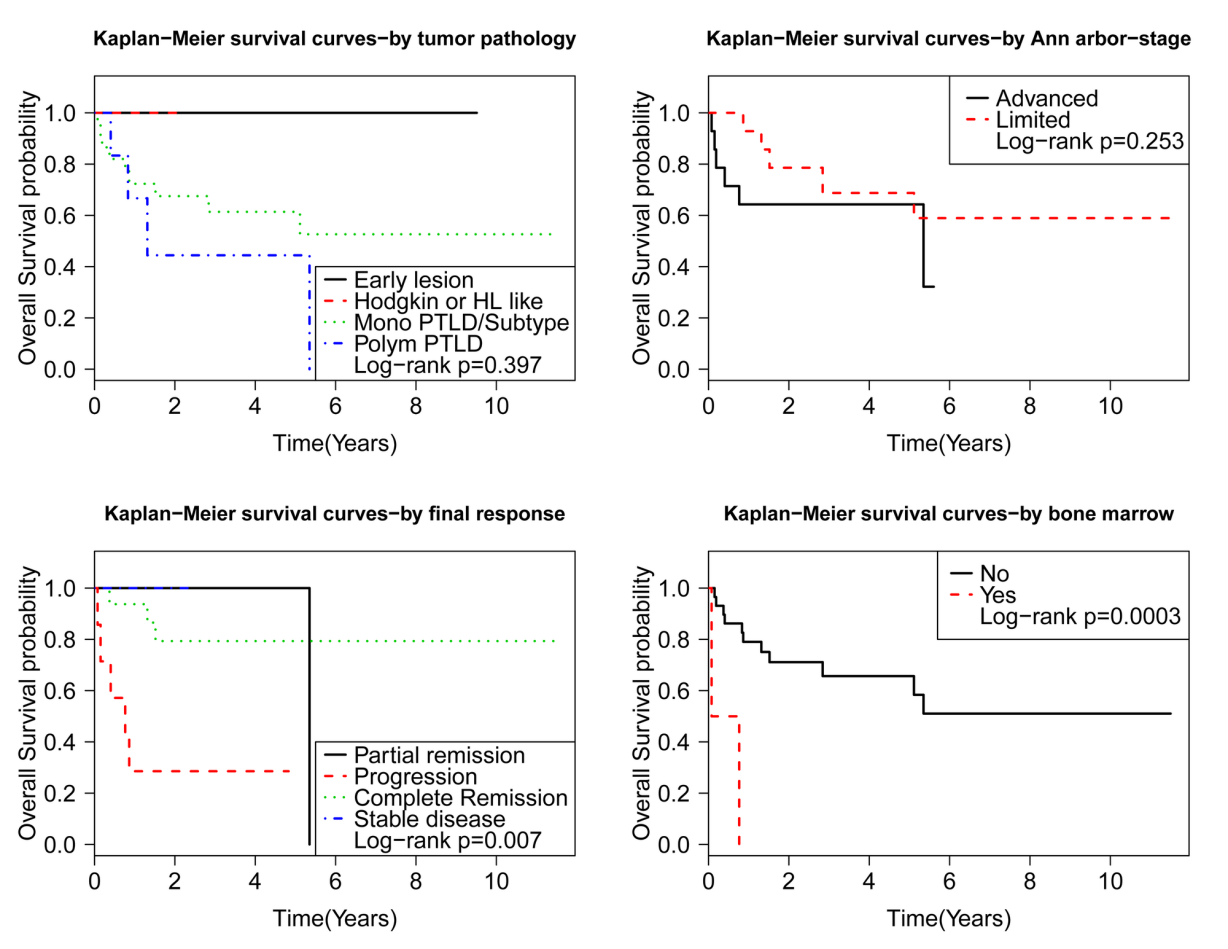
Kaplan-Meier (KM) analysis survival based on PTLD characteristics

Disease characteristics, including Ann Arbor stage, PTLD pathology subtype, CD20 status, EBER status, bulky disease, and central nervous system (CNS) involvement were not significant for survival. Bone marrow (BM) involvement had a statistically significant HR of 12.1 (95%CI 2.2-66.5, p=0.004). On a multi-variate analysis, based on the model selection criteria, BM involvement was the only significant factor that remained significant for survival (HR 11.4, p-0.0081). See Figure 2.

## Discussion

As the survival of patients after transplant has improved, we have seen PTLD in patients beyond the traditional early-onset and late-onset patient populations [21]. PTLD incidence has a bimodal distribution where there is an increase in incidence within one year of transplant and then another peak, which occurs around five years after transplant. Very-late onset PTLD is defined as PTLD occurring 10 or more years after the transplantation. Over the last two decades, there have been many retrospective studies from individual transplant centers and the factors affecting the survival of early-onset PTLD and late-onset PTLD are well-established but there is minimal scientific data for very-late onset PTLD. As prospective studies or clinical trials are difficult to perform in this rare entity, it becomes imperative that we retrospectively explore different subgroups of PTLD to identify different prognostic factors.

In this study, we explored this subgroup of 33 patients with very-late PTLD who were diagnosed between 10 and 18.4 years after their transplantation with a median of 13.3 years, thus indicating that this disease can develop at any point after transplantation, not just the first 10 years. While traditional disease characteristics, such as Ann Arbor stage, number of sites involved, performance status, PTLD pathology subtype, CD20 status, EBER status, bulky disease, and CNS involvement, have been found to be statistically significant in prognosticating early and late-onset PTLD, none of these disease characteristics were found to have a statistically significant impact on the prognosis of very-late onset PTLD in our analysis [19,27-28]. Instead, on univariate analysis, advanced age, male sex, type of active immunosuppressant drugs, past history of malignancy, bone marrow involvement, and response to PTLD treatment were significant while on multivariate analysis, only bone marrow involvement at the time of diagnosis was found to be the statistically significant prognostic marker. In fact, patients with advanced Ann Arbor stage had similar survival to those with a limited stage. There is enough scientific data to support that EBV plays a significant role in the pathogenesis of PTLD, but its role as a prognostic factor is questionable [6,24]. That significance appears to decrease with time, as the EBV status of the recipient is non-significant in the cases of very-late onset PTLD. Performance status is a known significant prognostic factor but was not found to have an effect on the survival of patients that presented with very-late onset PTLD.

Multiple previous studies have shown that different immunosuppressive agents also affect survival and, in fact, RIS is usually employed as the first strategy for the treatment of PTLD, either alone or in combination with chemoimmunotherapy [9,29]. While the reduction in immunosuppression helps with PTLD treatment by improved host immunity, it also increases the risk of allograft rejection and, sometimes, it is hard to achieve a delicate balance [29]. Our patient population developed PTLD more than 10 years of transplant, at which time, patients have been exposed to prolonged immunosuppression over the years. Various previous studies have explored the risk of PTLD with the use of induction immunotherapy and different immunosuppressive agents as maintenance immunosuppression but there is limited data on the impact of different immunosuppressive agents on the survival of patients with PTLD [5,30]. We found that the use of induction immunotherapy, previous episodes of acute rejections, or chronic rejection did not affect survival. Interestingly, the use of cyclosporine with azathioprine in combination at the time of diagnosis was a statistically significant worse prognostic factor (HR 5.7, 95% CI: 1.8-18.4, p= 0.003) while the use of tacrolimus at the time of PTLD diagnosis, either in combination or by itself, was associated with improved overall survival (HR of 0.13, 95%CI: 0.03-0.57, P= 0.007). This finding is interesting, as previous studies have shown that the use of tacrolimus as an immunosuppressive agent is associated with a five-fold increase in the risk of PTLD [30]. This finding suggests the role of immunosuppression over a prolonged time and choice of agents can affect the survival of PTLD significantly. It was interesting to see that after 10 years of transplant, the risk of allograft rejection and failure was so less. Of 33 patients, only one patient experienced acute rejection and, eventually, only two patients lost graft function but one was re-transplanted after PTLD treatment. This finding emphasizes that PTLD treatment likely does not pose a significant risk of graft rejection or failure in a patient who develop PTLD 10 years post-transplant. We also noticed that CD20 positivity or the use of rituximab or choice of different chemotherapeutic agents did not affect survival. However, we did find that the initial best response after the first treatment strategy was associated with survival. Patients who progressed with the first line of treatment had an HR of 6.3 (95% CI: 1.4 - 28.1, p = 0.015) and tend to do poorly even after other lines of treatment.

In summary, the data from our study shows that the traditional prognostic factors known for early or late PTLD are likely not applicable to this subgroup of very-late PTLD, as these patients are usually older and have been on relatively longer immunosuppression. After evaluating the prognostic factors of very late-onset PTLD, it appears that it is a third distinct subtype of PTLD.

Study limitations

This study is retrospective in nature and thus carries all the inherent limitations of a retrospective study. Although data collection was robust, there is always clinically relevant information that is not available in medical records. As such, we do note that the evidence from this study is not as rigorous as from a randomized controlled trial (RCT) but given the rarity of very-late PTLD, a retrospective analysis, as conducted by the researchers, is the most practical method of obtaining a clinically meaningful insight into the problem. Another limitation is the small number of patients, however, there is a very small subgroup of patients who have developed very-late PTLD when compared to all patients with PTLD (a very rare group in and of itself). Our literature search did not show any similar studies from the United States.

## Conclusions

As the survival of transplant patients improves, the incidence of PTLD diagnosed after many years of transplant is rising. Very-late onset PTLD is a minimally researched subtype of PTLD. We identified various prognostic factors in this subgroup that are different from the known prognostic factors for PTLD and should be taken into consideration for the clinical course and treatment of very-late onset PTLD.
